# *Pichia pastoris* Fep1 is a [2Fe-2S] protein with a Zn finger that displays an unusual oxygen-dependent role in cluster binding

**DOI:** 10.1038/srep31872

**Published:** 2016-08-22

**Authors:** Antimo Cutone, Barry D. Howes, Adriana E. Miele, Rossella Miele, Alessandra Giorgi, Andrea Battistoni, Giulietta Smulevich, Giovanni Musci, Maria Carmela Bonaccorsi di Patti

**Affiliations:** 1 Dip. Scienze Biochimiche ‘A. Rossi Fanelli’, Sapienza Università di Roma, Roma, Italy; 2 Dip. Chimica ‘Ugo Schiff’, Università di Firenze, Sesto Fiorentino (FI), Italy; 3 Dip. Biologia, Università di Roma Tor Vergata, Roma, Italy; 4 Dip. Bioscienze e Territorio, Università del Molise, Pesche, Italy

## Abstract

Fep1, the iron-responsive GATA factor from the methylotrophic yeast *Pichia pastoris,* has been characterised both *in vivo* and *in vitro*. This protein has two Cys_2_-Cys_2_ type zinc fingers and a set of four conserved cysteines arranged in a Cys-X_5_-Cys-X_8_-Cys-X_2_-Cys motif located between the two zinc fingers. Electronic absorption and resonance Raman spectroscopic analyses in anaerobic and aerobic conditions indicate that Fep1 binds iron in the form of a [2Fe-2S] cluster. Site-directed mutagenesis shows that replacement of the four cysteines with serine inactivates this transcriptional repressor. Unexpectedly, the inactive mutant is still able to bind a [2Fe-2S] cluster, employing two cysteine residues belonging to the first zinc finger. These two cysteine residues can act as alternative cluster ligands selectively in aerobically purified Fep1 wild type, suggesting that oxygen could play a role in Fep1 function by causing differential localization of the [Fe-S] cluster.

The regulation of transition metals is critical for all living cells due to the dual nature of these elements, which are essential for function but can become extremely toxic if their levels are not tightly controlled inside the cell. Iron-responsive transcriptional repressors, which bind to a 5′-(A/T)GATAA-3′ sequence and are therefore defined as GATA factors, have been identified in the fission yeast *Schizosaccharomyces pombe* and in various fungi such as *Aspergillus nidulans, Neurospora crassa, Penicillium chrysogenum, Ustilago maydis* and others [ref. 1 and references therein]. A similar mechanism of down-regulation of genes involved in reductive and non-reductive iron uptake is effective also in the pathogenic yeast *Candida albicans* through the activity of the GATA factor Sfu1^2^. We have demonstrated that a GATA-type transcriptional repressor regulates high affinity iron uptake also in the methylotrophic yeast *Pichia pastoris*^3^, indicating that this is a widely adopted mode of regulation.

Iron-responsive fungal GATA factors typically possess an N-terminal DNA-binding domain and a C-terminal repression domain. The N-terminal domain is characterized by the presence of two zinc fingers of the Cys_2_-Cys_2_ type and a set of four highly conserved cysteines arranged in a Cys-X_5_-Cys-X_8_-Cys-X_2_-Cys motif located between the two zinc fingers. The C-terminal domain shows very limited sequence similarity among different iron-responsive GATA factors; it has been shown to be involved in dimerization and recruitment of the co-repressors Tup11 and Tup12, at least in the cases of *S. pombe* Fep1 and *C. albicans* Sfu1^4^,^5^.

The molecular mechanism of iron sensing by fungal GATA factors is still unclear; evidence that the Cys-X_5_-Cys-X_8_-Cys-X_2_-Cys motif is involved in this process has been obtained by replacing two or all four cysteines with either serine or alanine. These mutations inactivate the factor by decreasing its affinity for DNA^6^,^7^,^8^. A role for the Cys-rich region in iron binding has been suggested for Sre, the GATA factor involved in siderophore biosynthesis in *N. crassa*^6^. More recently, an analysis of the metal content of *Histoplasma capsulatum* Sre1 has indicated that this GATA factor binds a substoichiometric amount of iron^8^. Finally, *S. pombe* Fep1 has been recently reported to bind iron^9^. However, a detailed biochemical characterization of how iron is bound to an iron-responsive GATA factor is still lacking.

We have functionally and spectroscopically characterized Fep1, the iron-responsive GATA factor of the methylotrophic yeast *Pichia pastoris*, and we demonstrate that iron is bound to the protein as a [2Fe-2S] cluster. Site-directed mutagenesis analysis on aerobically and anaerobically purified Fep1 indicates that cysteine residues other than those belonging to the Cys-X_5_-Cys-X_8_-Cys-X_2_-Cys motif can be involved in cluster binding in an oxygen-dependent fashion.

## Results and Discussion

### Functional complementation of the P. pastoris fep1∆ strain by Fep1

We have already reported the cloning of the region between the two zinc finger motifs (highly conserved in other iron-responsive fungal GATA factors) of *P. pastoris* Fep1^3^. We then cloned the entire coding sequence of Fep1 (337 amino acids) plus 415 bp upstream of the ATG codon and 250 bp downstream of the stop codon (GenBank accession number AM503349) by inverse PCR on genomic DNA. Our sequence is identical to that found as a result of the *Pichia* genome sequencing project of strain GS115^10^, which was made publicly available after completion of our work.

**Figure 1:**
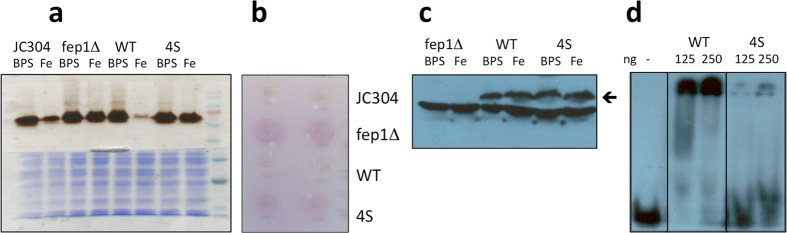
Complementation of *P. pastoris* strain *fep1Δ* by Fep1 WT but not by Fep1 4S. (**a**) Non-denaturing SDS-PAGE analysis of Fet3 oxidase activity of JC304, *fep1Δ*, Fep1 WT and 4S grown in YPD + BPS 80 μM or YPD + FeCl_3_ 100 μM. The upper part of the gel is stained with *o*-dianisidine to show Fet3 oxidase activity; the lower part of the gel is stained with Coomassie Blue to show total protein content of samples loaded on the gel. (**b**) *In vivo* iron-reductase assay of JC304, *fep1Δ*, Fep1 WT, Fep1 4S. Iron-reductase activity was assayed by placing a nitrocellulose filter directly onto colonies grown on MD + FeCl_3_ 200 μM plates and overlaying with a solution of 1 mM ferrozine, 1 mM FeCl_3_ in 50 mM sodium acetate, pH 5. (**c**) Western blot of *fep1Δ* and Fep1 WT and 4S grown in YPD + BPS 80 μM or YPD + FeCl_3_ 100 μM. The arrow indicates recombinant Flag-tagged Fep1. (**d**) EMSA analysis of DNA binding by MBP-Fep1 (41–192) WT and 4S. All panels show representative results of experiments run at least 2 times.

Recombinant Fep1 WT with a C-terminal Flag tag expressed in a *fep1∆* strain restored the iron-dependent regulation of two systems under the physiological control of Fep1, namely the ferroxidase Fet3 and the iron-reductase complex. In fact, cells grown in iron limiting conditions (i.e, in the presence of the iron chelator BPS) showed high Fet3 oxidase activity, while those grown under conditions of iron abundance showed very low oxidase activity (Fig. 1a). Iron-reductase activity can be readily observed as a purple color on colonies overlaid with a nitrocellulose filter soaked in a solution containing Fe^3+^ and ferrozine^3^; Fig. 1b shows that colonies of *fep1Δ* expressing recombinant Fep1 recovered the wild type phenotype.

**Figure 2:**
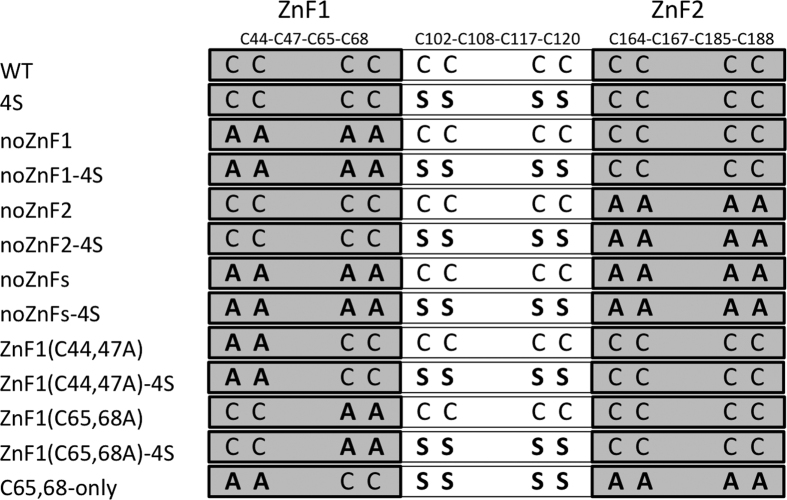
Scheme of Fep1 cysteine mutants produced and characterized in this work. The bold font denotes mutated cysteines.

To investigate the functional role of the four highly conserved cysteine residues of the Cys-X_5_-Cys-X_8_-Cys-X_2_-Cys motif between the two zinc fingers, we produced a mutant in which all four cysteines (Cys102, Cys108, Cys117, Cys120) were replaced with serine. The mutant was named 4S. Fig. 2 shows a scheme of all Fep1 mutants used in this study, together with their acronyms.

Functional complementation assays on Fep1 4S showed high expression of Fet3, both in limiting or abundant iron conditions (Fig. 1a), and high iron-reductase activity (Fig. 1b), indicating that the mutant failed to regulate its targets and conferred a phenotype very similar to the *fep1Δ* strain. These results indicate that the four conserved cysteine residues are needed for Fep1 to be functional and to respond to intracellular iron changes. Western blot analysis confirmed expression of Fep1 WT and of the 4S mutant (Fig. 1c): the recombinant factor is clearly visible as a band with M_r_ slightly above 40 kDa in the transformed strains when compared to the *fep1Δ* strain, although there is high cross-reactivity of the monoclonal anti-Flag antibody.

Fluorescence microscopy of a GFP-Fep1 fusion demonstrated that the WT and 4S mutant proteins were constitutively localized in the nucleus of cells grown in the presence of BPS or of excess iron (Fig. S1). This result indicates that iron-mediated regulation by Fep1 does not occur through nucleus-cytoplasm protein translocation, consistent with the analogous cases of *S. pombe* Fep1^7^ and *C. albicans* Sfu1^5^.

### Biochemical characterization of Fep1

Recombinant full-length Fep1 WT and 4S expressed in *E. coli* and purified by cation exchange chromatography were extremely prone to degradation, with multiple lower molecular weight bands visible in SDS-PAGE (Fig. S2a). We had previously produced a stable MBP-Fep1 fusion protein^3^, where the maltose binding protein was fused to residues 41–192 of Fep1, which include the two zinc finger motifs and the intervening Cys-rich sequence. In this study we produced the corresponding MBP-Fep1 4S mutant to test its DNA binding ability. As a probe for the DNA band-shift assay we used an oligonucleotide containing the GATA sequence of the promoter region of FET3^3^. As expected, MBP-Fep1 4S showed a decreased affinity for DNA compared to WT (Fig. 1d). This result is in line with analogous EMSA analyses performed on *S. pombe* Fep1^7^, *N. crassa* Sre^6^ and *H. capsulatum* Sre1 cysteine mutants^8^, which invariably showed at least a 2–2.5-fold lower affinity for DNA compared to WT. Finally, we produced Fep1 WT and 4S as a truncated, flagged version corresponding to the DNA-binding domain comprising residues 1–208. Expression and purification were successful, both Flag-Fep1 (1–208) WT and 4S were stable and could be obtained with high purity (Fig. S2b). Mass spectrometry analysis confirmed the integrity of the two proteins.

All purified full-length and truncated Fep1 proteins, both WT and 4S mutant, were stably reddish-brown (Fig. S2c). This is not surprising, at least for the WT protein, since the four conserved ‘central’ cysteines in the region between the two zinc finger motifs could form a binding site for iron, giving rise to an optically active Fe-S cluster.

**Figure 3:**
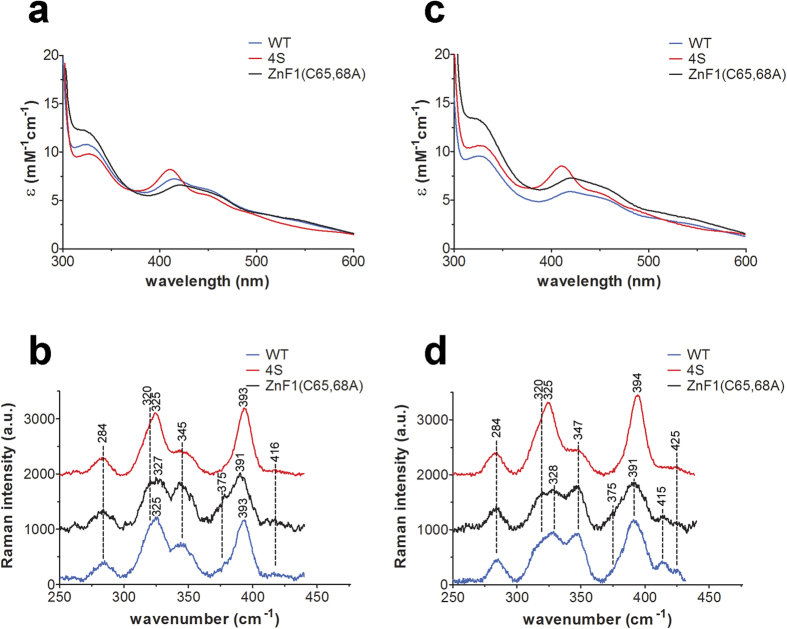
Electronic absorption and resonance Raman spectra of Fep1. Comparison of the electronic absorption and RR spectra of Fep1 (1–208) WT, 4S and ZnF1(C65,68A) obtained aerobically (**a**,**b**) and anaerobically (**c**,**d**) at room temperature. Electronic absorption molar extinction coefficients (ε) are based on the concentration of iron. RR experimental conditions: excitation wavelength 413.1 nm; laser power at the sample 50 mW; integration time of each spectrum 10 min; WT, average of 6 spectra (**b**) and 12 spectra (**d**); 4S, average of 6 spectra (**b**,**d**); ZnF1(C65,68A), average of 16 spectra (**b**) and 12 spectra (**d**). The RR intensities are normalised to that of the band at 284 cm^−1^.

The absorption spectra obtained for aerobically prepared full-length Fep1 WT and 4S were qualitatively indistinguishable from the spectra of the corresponding DNA-binding domains (1–208) (Fig. S3 and Fig. 3a, respectively). Hence, the partial degradation of the full-length proteins does not significantly affect the geometry of the iron-binding site. However, since time and/or storage further affected the integrity of the full-length proteins, all subsequent analyses were carried out on the stable Fep1 (1–208) truncated derivatives. The optical spectra of Fep1 (1–208) WT and 4S (Fig. 3a) show two main absorption features: a shoulder at 325 nm and a peak at 410–420 nm with a shoulder at 455 nm. In particular, the WT protein displays a peak maximum at 413 nm, whereas a more pronounced peak is observed for the 4S mutant at 410 nm, endowing the two proteins with a different shade of red-brown color (Fig. S2c). These features are broadly characteristic of S **→** Fe charge transfer bands of [2Fe-2S] clusters^11^,^12^,^13^,^14^,^15^,^16^. The molar extinction coefficients calculated on a *per* iron basis are in the range reported for [2Fe-2S] clusters (between 7 and 11 mM^−1^ cm^−1^, i.e. 3.5–5.5 mM^−1^ cm^−1^ *per* Fe)^11^,^12^,^13^,^17^.

Identification of a [2Fe-2S] cluster solely on the basis of the optical absorption spectrum can be ambiguous. Resonance Raman (RR) spectroscopy of [Fe-S] proteins in the Fe-S stretching region (200–450 cm^−1^) provides instead a powerful means to distinguish the type and coordination of [Fe-S] clusters: the frequencies of the predominantly Fe-S^t^ (Cys) B_3u_^t^ and Ag^t^ stretching modes of [2Fe-2S] clusters provide a direct indication of whether the cluster has a complete cysteine coordination^18^,^19^ or an incomplete cysteine coordination, where one thiol ligand can be substituted by an oxygen^19^,^20^ or an imidazole group^21^,^22^. In general, compared to clusters with complete thiol coordination, an oxygen ligand gives rise to an upshift of the B_3u_^t^ and Ag^t^ modes on the basis of the mass difference between S and O. Accordingly, for a [2Fe-2S] cluster with partial non-cysteinyl ligation, these bands shift up (ca. 295 and 353-356 cm^−1^, respectively) compared to those of the all-cysteine-ligated [2Fe-2S] clusters in ferredoxins (280–290 and 326–340 cm^−1^, respectively). In the case of His coordination, the intense B_3u_^t^ mode at ca. 280–290 cm^−1^ is replaced by weak Fe-N modes in the 250–300 cm^−1^ region.

The RR spectra of aerobically prepared Fep1 (1–208) WT and 4S obtained using 413.1 nm excitation reveal Fe–S stretching modes at the same frequencies in both cases (284, 320, 325, 345, 393 cm^−1^) (Fig. 3b). The vibrational frequencies are similar to those of structurally characterized [2Fe-2S]^2+^ ferredoxins with complete cysteinyl cluster ligation and are readily assigned under D_2h_ symmetry to vibrational modes of the Fe2S^b^2S^t^4 unit (S^b^ = bridging S and S^t^ = terminal or cysteinyl S) by direct analogy with the well-characterized [2Fe-2S] ferredoxins, which have been assigned via isotope shift and model studies^18^,^23^. The assignments of the Fe-S stretching frequencies observed for Fep1 WT and 4S are reported in Table S1. There is some slight uncertainty regarding the assignment of the band at 345 cm^−1^. We have assigned this band to the B_1u_^t^/B_2g_^t^ modes, which predominantly involve Fe-S^t^ stretching and are only enhanced for excitation at 406/413 nm^18^; however we cannot exclude that it derives from a B_3u_^b^ mode that becomes significantly more enhanced for visible wavelength excitation^23^. This uncertainty is due to the very strong fluorescence observed for the Fep1 proteins at visible wavelength excitation, which prevented a thorough characterization of the excitation profile. The band at 375 cm^−1^ observed in the WT spectrum, by analogy with a band observed at ca. 370 cm^−1^ in oxidized putidaredoxin only for 406.7 nm excitation^18^, is tentatively assigned to an internal deformation mode involving the amide N of one or more of the coordinated cysteines. Although the spectra of WT and 4S are very similar, slight differences in relative intensity of the bands at 320, 345 and 375 cm^−1^ can be noted. This is likely due to the fact that the aerobically purified WT protein is a mixture of two forms (see below).

The iron content of freshly purified Fep1 (1–208) WT and 4S was about 0.2 Fe/protein (Table S2), probably because overexpression of the recombinant protein saturates the cellular iron-sulfur cluster assembly machinery. Acid-labile sulfur was detected in both proteins, further confirming the presence of an iron-sulfur cluster. Zinc content was about 1.5 atoms/protein, similar to the value of 1.6 reported for *H. capsulatum* Sre1^8^. In fact, lower than expected zinc content values are not infrequent in recombinant zinc finger proteins, particularly those with multiple ZnFs. Iron and acid-labile sulfur contents are clearly suboptimal; however, [Fe-S] clusters can often be reconstituted by addition of inorganic iron and sulfide in the presence of a reducing agent^23^,^24^. Thus, reconstitution of Fep1 by aerobic addition of FeCl_3_ and Na_2_S in the presence of TCEP was attempted. The optical spectra of reconstituted Fep1 (1–208) WT and 4S showed a substantially increased intensity of the bands, yet no apparent change in shape, indicating that the iron cluster could be reconstituted in these conditions (Fig. S4). The reconstituted cluster was stable in the presence of oxygen for at least a few hours. Iron stoichiometry after reconstitution was close to 1 Fe/protein monomer (Table S2), in line with that reported for *H. capsulatum* Sre1 WT, which was reddish-brown and was demonstrated to bind 0.5–1 Fe/protein monomer by ICP-AES^8^. Acid-labile sulfur also increased to about 1 S/protein monomer, while zinc content decreased to about 1 Zn/protein monomer. The reconstituted 4S mutant had a somewhat lower labile sulfur content, compared to WT. One possible explanation is that part of the iron after reconstitution is not in the form of an iron-sulfur cluster, but rather it is aspecifically bound to the protein, which would distort the Fe/S ratio (in fact this is also partially true for the WT protein). This would imply that, for unknown reasons, the 4S mutant is more impervious to reconstitution than the WT protein.

**Figure 4:**
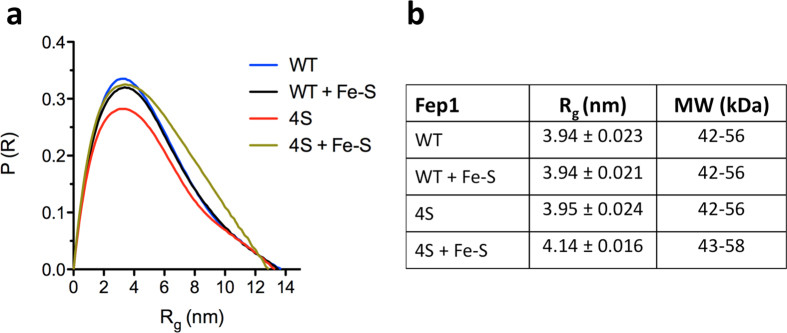
SAXS analysis of Fep1. (**a**) Normalized electron-pair distribution function profiles of Fep1 (1–208) WT and 4S as-purified and after reconstitution. The profile of reconstituted 4S indicates mild (3–5%) aggregation, which is not observed in the other samples. (**b**) Radii of gyration and molecular weight of Fep1 (1–208) WT and 4S determined by SAXS.

Size exclusion chromatography suggested that Fep1 (1–208) WT and 4S are both dimeric. SAXS analysis confirmed that Fep1 (1–208) WT and 4S are indeed dimeric, independently of the amount of [2Fe-2S] cluster bound by the protein (Fig. 4). The radius of gyration, a measure of the overall molecular size, of Fep1 (1–208) WT and 4S as-purified and after reconstitution was determined from the electron-pair distribution function. The radius of gyration and the shape of the electron-pair distribution function are similar for WT and 4S and do not change significantly after reconstitution (Fig. 4). Estimated molecular weights from *ab initio* calculation with the ATSAS suite^25^ are indicative of a dimer.

### Identification of Fep1 (1–208) [2Fe-2S] cluster ligands

The optical spectrum of Fep1 WT is similar to that of *N. crassa* Sre WT^6^. However, mutants of the four central Cys of *N. crassa* Sre were reported to be colorless^6^ and those of *H. capsulatum* Sre1 were claimed to show a yellow color and did not bind an appreciable amount of iron^8^. Intriguingly, the electronic absorption and RR spectra of the Fep1 4S mutant have features consistent with the presence of a [2Fe-2S] cluster, implying that the four cysteines of the Cys-X_5_-Cys-X_8_-Cys-X_2_-Cys motif may not be the only ones involved in iron binding. Alternatively, other groups could replace their role as iron ligands in their absence (i.e. in the 4S mutant).

The RR spectra indicate that the [2Fe-2S] cluster in the 4S mutant still possesses full cysteinyl ligation. Therefore, although serinate ligation of Fe-S clusters has been described for different protein mutants^26^,^27^,^28^, we can exclude that this is the case for Fep1 4S. Glutathione can be a ligand for some Fe-S clusters, as reported for yeast Fra2-Grx3 and human BolA2-Grx3 heterodimers^11^,^29^, yet no glutathione was detected in recombinant Fep1 (1–208) WT nor in all the mutants.

The only other Cys in the amino acid sequence of Fep1 belong to the two Zn fingers. One possibility is that one or more of these Cys form a sort of ‘rescue’ site in the 4S mutant. To address this issue a set of mutants targeting the cysteine residues belonging to the two Zn finger motifs were produced and analyzed. For each Zn finger, all four cysteines were replaced with alanine to produce single (“noZnF1” and “noZnF2”) or double (“noZnFs”) Zn finger mutants. All Zn finger mutations were introduced also in a 4S background (see Fig. 2 for nomenclature) and the mutants were expressed in *E. coli* and purified with the same protocol as the WT protein.

**Figure 5:**
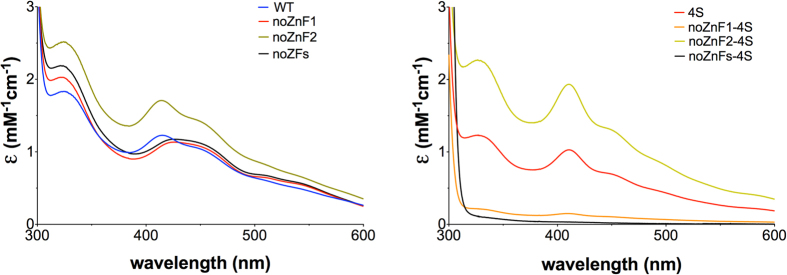
Absorption spectra of Fep1 (1–208) Zn finger mutants aerobically purified. Molar extinction coefficients (ε) are expressed per Fep1 monomer.

The optical spectra of the aerobically prepared Fep1 Zn finger mutants as-purified are shown in Fig. 5. Strikingly, the noZnF1 and noZnFs mutants, lacking the first Zn finger, showed altered absorption spectra compared to WT that, however, are still consistent with a [2Fe-2S] cluster. When these mutations were inserted in the 4S frame, i.e. when the four ‘central’ cysteines were also missing, both mutants were practically devoid of iron spectroscopic features. On the other hand, the second Zn finger appears to have no influence on iron binding, as the noZnF2 mutants had spectroscopic features identical to those of the parental WT or 4S proteins. These results strongly suggest that [2Fe-2S] binding requires the presence of at least one of two elements: the first Zn finger and the Cys-X_5_-Cys-X_8_-Cys-X_2_-Cys motif; each element imparts a peculiar spectroscopic signature to the iron site and iron binding is absent when both elements are mutated. As expected, iron and acid-labile sulfur content of the mutants was similar to those obtained for as-purified Fep1 (1–208) WT and 4S, except for the noZnF1-4S and noZnFs-4S proteins that bound less than 0.02 Fe/protein (Table S3).

To dissect the contribution of ZnF1 cysteines (Cys44, Cys47, Cys65, Cys68) to cluster binding, a further set of mutants was produced and characterized. Mutants lacking Cys44 and Cys47 —ZnF1(C44,47A) and ZnF1(C44,47A)-4S — were similar to 4S, indicating that the first two cysteines do not participate in cluster ligation (Fig. S5). On the other hand, ZnF1(C65,68A) and ZnF1(C65,68A)-4S mutants with Cys65 and Cys68 changed to Ala recapitulate the spectral properties of the respective noZnF1 proteins (Fig. S5), demonstrating that these two Cys are involved in [2Fe-2S] cluster binding. The absorption spectrum of ZnF1(C65,68A) is also shown for comparison with WT and 4S in Fig. 3a. The RR spectrum of the ZnF1(C65,68A) mutant (Fig. 3b) is very similar to WT; its spectrum differs primarily in the increased relative intensity of the bands at 320, 345 and 375 cm^−1^. Hence, as for the WT protein, it is consistent with a [2Fe-2S] cluster characterized by complete cysteinyl ligation. The change in relative intensity of the band at 345 cm^−1^ between aerobically prepared ZnF1(C65,68A) and 4S (Fig. 3b) may be simply due to a change in intensification of the closely positioned modes B_1u_^t^/B_2g_^t^ and B_3u_^b^ in the two proteins. Namely, that in one case the band should be assigned to B_1u_^t^/B_2g_^t^ modes, whereas in the other case to B_3u_^b^. Alternatively, consistent with the variations in their electronic absorption spectra, the variations in the relative intensities of some RR bands between ZnF1(C65,68A) and 4S may be related to differences in their S ***→*** Fe charge transfer transitions, as has been reported for some ferrodoxins in the same experimental conditions^18^.

A final mutant named C65,68-only that lacked all cysteines except Cys65 and Cys68 in ZnF1 was produced. This mutant showed an optical spectrum identical to that of 4S (Fig. S6), definitely demonstrating that Cys65-Cys68 are sufficient for cluster binding.

Therefore, these results unequivocally show that in the 4S mutant the [2Fe-2S] cluster is bound by Cys65-Cys68 belonging to zinc finger ZnF1. Conversely, in Fep1 mutants lacking these two residues (noZnF1 or ZnF1(C65,68A)) the cluster can only be ligated by the central cysteines. The two clusters have clearly distinguishable electronic absorption and RR spectroscopic signatures.

The dimeric nature of the protein suggests that cluster ligands may originate from the two subunits making up the dimer; this would be an obligate choice in the case of binding by Cys65-Cys68, and the [2Fe-2S] cluster would necessarily bridge the Fep1 dimer. In the case of binding by Cys102-Cys108-Cys117-Cys120, we are presently unable to define precisely if all four or only some of the cysteines are ligands for the cluster and whether all four ligands originate from the same monomer or not. Studies are ongoing to address these issues.

### A role for oxygen in [2Fe-2S] cluster binding at ZnF1

Since the characterization reported above was carried out in air, the question arose whether we were actually observing degradation products of a native cluster, with a [4Fe-4S] cluster being a likely candidate. Therefore, we prepared anaerobic purifications of Fep1 WT, 4S and ZnF1(C65,68A), and analyzed the proteins by keeping the samples strictly in the absence of oxygen. The optical and RR spectra of the anaerobically purified proteins are shown in Fig. 3c,d. It is clear that both mutants exhibit spectroscopic properties very similar to those of the aerobically purified proteins. Conversely, anaerobically purified Fep1 WT showed optical and RR spectra very similar to those of the ZnF1(C65,68A) mutant, but different from those obtained for the aerobically purified protein (Fig. 3a,b and Table S1). The anaerobic purifications were repeated twice, with identical results. The RR spectra of anaerobically purified Fep1 WT and the ZnF1(C65,68A) mutant obtained using 413.1 nm excitation are essentially identical and display Fe-S stretching frequencies at 284, 320, 328, 347, 391 and 415 cm^−1^. The RR spectrum of 4S shows marked differences in relative intensity of the bands at 328 and 347 cm^−1^ and slight differences in frequencies of the bands at 328 and 391 cm^−1^ compared to WT. As noted above for the RR spectra of the aerobically purified proteins, the marked change in relative intensity of the band at 347 cm^−1^ between anaerobically purified WT/ZnF1(C65, 68A) and 4S (Fig. 3d) may possibly be due to a change in intensification of the closely positioned modes B_1u_^t^/B_2g_^t^ and B_3u_^b^ in the two proteins.

It is noteworthy that the similarity of the electronic absorption and RR spectra of the anaerobically and aerobically prepared mutants indicates that the coordination and structural characteristics of the two [2Fe-2S] clusters (that bound to the central Cys residues and that on ZnF1) are not significantly sensitive to the presence of oxygen. It is noted that there is no evidence for the presence of a [4Fe-4S] cluster in any of the anaerobically or aerobically prepared WT or mutant samples.

**Figure 6:**
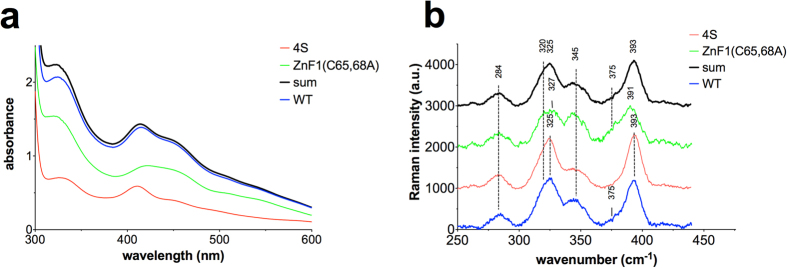
Aerobically purified Fep1 WT is a mixture of two forms with different [2Fe-2S] cluster localization. Absorption (**a**) and RR spectra (**b**) of Fep1 obtained by 1:1 sum of equimolar (in Fe) 4S and ZnF1(C65,68A) mutants compared to the spectrum of Fep1 WT. The WT and mutant spectra shown correspond to those of Fig. 3a,b.

The close similarity of the electronic absorption and RR spectra of the anaerobically prepared WT and ZnF1(C65,68A) mutant indicates that the anaerobically prepared WT is characterized by a [2Fe-2S] cluster positioned centrally between the two zinc fingers. This behaviour contrasts with WT prepared aerobically, that appears to be a mixture of two forms: one where the [2Fe-2S] cluster is bound by Cys102-Cys108-Cys117-Cys120 and one where the cluster ligands are Cys65-Cys68. This hypothesis is supported by the fact that the optical and RR spectra of aerobically purified Fep1 WT can be reconstructed by summing the spectra of any mutant lacking Cys65-68 (where the cluster is bound by the four central Cys) plus any mutant lacking the four central Cys (where the cluster is bound by Cys65–68). As an example, Fig. 6a,b shows the sum of the absorption and RR spectra of equimolar (in Fe) ZnF1(C65,68A) plus 4S. The vast majority of aerobically purified Fep1 (1–208) WT samples showed spectra as those depicted in Fig. 3a (Fig. S7, thick blue spectrum), indicative that in the presence of oxygen the protein is a mixture of the two forms. In some rare cases the protein had a spectrum with a less resolved peak at 413 nm (Fig. S7, WT-a), similar to that of the anaerobically purified protein, or one exhibiting a close correspondence with that of the 4S derivative (Fig. S7, WT-b). In one extreme case, Fep1 (1–208) WT had apparently fully switched to a 4S-like form (Fig. S7, WT-c). It is noteworthy that the bacterial lysate of this specific preparation showed the typical WT shade of red-brown color, but the purified protein had changed to 4S-type optical spectra. The spectral lineshape of any sample of Fep1 WT could be reconstructed by varying the fractional contribution of the two component spectra (ZnF1(C65,68A) and 4S). This finding explains the above-mentioned slight differences in relative intensity of RR bands between aerobically purified WT and 4S. In fact, the spectrum of aerobically purified Fep1 WT is a composite of different proportions of a 4S-type and a ZnF1(C65,68A)-type spectrum, depending on the relative amount of each form in the mixture. Therefore, the observed variations in the absorption lineshape and in the relative intensity of the RR bands will simply reflect the relative proportions of the two forms.

**Figure 7:**
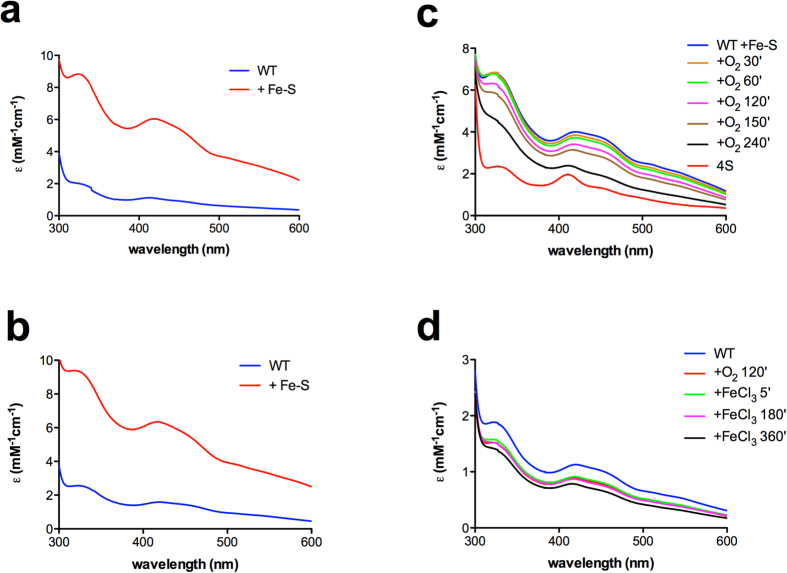
Exposure to oxygen changes the localization of the [2Fe-2S] cluster of Fep1 WT. Absorption spectra of Fep1 (1–208) WT purified aerobically (**a**) and anaerobically (**b**) before and after anaerobic reconstitution with 3-fold excess of FeCl_3_ and Na_2_S in the presence of 1 mM TCEP. Molar extinction coefficients (ε) are expressed per Fep1 monomer. (**c**) Time-course of absorption spectra of anaerobically reconstituted Fep1 (1–208) WT after exposure to air for the times indicated; for comparison an arbitrarily scaled spectrum of the 4S mutant is also shown. (**d**) Absorption spectra of anaerobically purified Fep1 (1–208) WT 260 μM after exposure to air and addition of 5 μM FeCl_3_.

Metal content (Fe/S and Zn) of anaerobically purified Fep1 was similar to that of the aerobically purified protein (Table S2). Anaerobic reconstitution of Fep1 (1–208) WT yielded optical spectra consistent with a [2Fe-2S] cluster positioned centrally between the two ZnFs (Fig. 7), irrespective of whether the protein had been anaerobically or aerobically purified. This result indicates that in the absence of oxygen, Cys65-Cys68 are not involved in binding of a [2Fe-2S] cluster. Exposure of anaerobically reconstituted Fep1 (1–208) WT to air led to time-dependent changes in the optical spectra consistent with a shift of the cluster from the central Cys to ZnF1 in a few hours, leading to a mixture of the two forms (Fig. 7c). Comparison with the 4S spectrum shows that after 240 min the final state, corresponding to the cluster located exclusively on ZnF1, has not been achieved. This result is consistent with the observation that some of the [2Fe-2S] cluster is still located in the region between the two ZnFs in aerobically purified (and reconstituted) Fep1 WT. On the other hand, the anaerobically purified protein (not reconstituted) was stable in the presence of oxygen, unless a small amount of FeCl_3_ was added (Fig. 7d), suggesting that the situation may be more complex and also other factors may contribute to accelerate the process.

These findings suggest that oxygen could modulate the function of Fep1 by causing an alternative localization of the [2Fe-2S] cluster at the ZnF1 site rather than at the central site. In fact, we predict that when the cluster is bound by Cys65-Cys68, the protein is inactive as observed for the 4S mutant *in vivo* (i.e., it has decreased affinity for DNA and it is unable to repress its target genes, see Fig. 1); when the cluster is bound by Cys102-Cys108-Cys117-Cys120 the conformation of Fep1 is such that the protein is active. Accordingly, the ZnF1(C65,68A) mutant should be constitutively active. However, although GFP-Fep1 ZnF1(C65,68A) was correctly localized to the nucleus, the mutant failed to restore regulation of its targets (Fig. S8). Lack of activity is most likely due to the fact that ZnF1 is disrupted in the mutant. In support of this assumption, it has been reported that both ZnF1 and ZnF2 are required for iron-mediated repression of *S. pombe* Fep1 or *N. crassa* Sre targets *in vivo*^7^,^30^.

From a structural point of view, this model implies that Fep1 must be extremely flexible and suggests a regulatory role of ZnF1 through its ability to supply ligands to a [2Fe-2S] cluster. Interestingly, it has been demonstrated that in *S. pombe* Fep1 ZnF2 is essential for DNA binding *in vitro*, while ZnF1 modulates DNA binding affinity enhancing it 5-fold^7^. In the aerobically reconstituted protein the zinc content decreases from about 1.4 to about 1 atom/protein monomer (Table S2), consistent with the possibility that Cys65-Cys68 may switch from zinc to [2Fe-2S] cluster binding, causing loss of zinc from ZnF1. In contrast, the zinc content of anaerobically reconstituted Fep1 appeared not to decrease significantly compared to the non-reconstituted sample (Table S2), in line with the location of the [2Fe-2S] cluster at the central Cys.

**Figure 8:**
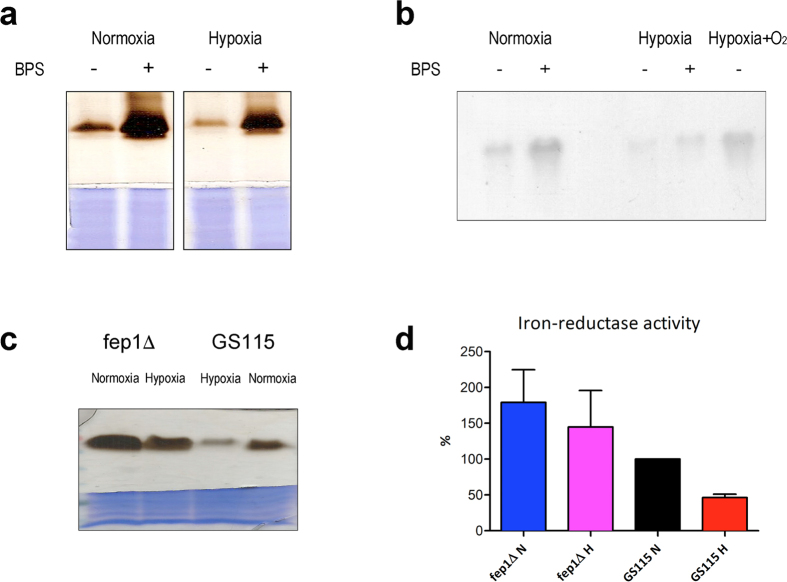
Fep1 activity is modulated by oxygen. Fep1-dependent expression of Fet3 and of the iron-reductase is decreased in hypoxia. (**a**,**b**) *P. pastoris* strain GS115 was grown for 18 hours in YPD −/+ BPS 80 μM in normoxia (40 ml culture in 250 ml flask) or in hypoxia (40 ml culture in 50 ml tube). (**a**) Non-denaturing SDS-PAGE analysis of Fet3 oxidase activity. The upper part of the gel is stained with *o*-dianisidine to show Fet3 oxidase activity; the lower part of the gel is stained with Coomassie Blue to show total protein content of samples loaded on the gel. (**b**) Northern blot analysis of FET3 expression. Total RNA (10 μg) from the same cells shown in panel a was probed with a 500-bp FET3 fragment labelled with ^32^P, as described^36^. Sample ‘Hypox + O_2_’ was transferred to a 250 ml flask and grown for 45 min after 18 hours in hypoxia. (**c**,**d**) *P. pastoris* strains GS115 and *fep1∆* were grown for 18 hours in YPD in normoxia (40 ml culture in 250 ml flask) or in hypoxia (40 ml culture in 50 ml tube). (**c**) Non-denaturing SDS-PAGE analysis of Fet3 oxidase activity as in panel a. (**d**) Iron-reductase activity (n = 4). Panels a–c show representative results of experiments run at least 3 times.

GATA-type Zn fingers belong to the treble clef family^31^; by analogy with mammalian GATA factors Cys65-Cys68 would be located at the first turn of the α-helix that together with a zinc knuckle supplies the four ligands in treble clef zinc fingers^31^. Our current hypothesis is that in response to an as yet unknown signal (either O_2_-dependent or O_2_-derived) this helix (that is involved in DNA binding and recognition in mammalian GATA factors^32^) could move away making Cys65-Cys68 available as [2Fe-2S] cluster ligands and, hence, modulate the affinity of Fep1 for DNA. Oxygen would thus represent a second modulator of Fep1 function: in the presence of oxygen a cluster can be assembled at the ZnF1 site and Fep1 activity would be impaired, allowing partial derepression of the iron regulon. To verify this prediction, GS115 cells were grown under hypoxic conditions and the expression levels of Fet3 were analyzed (Fig. 8a,b). Indeed, lower levels of Fet3 oxidase activity and mRNA were evident in hypoxic cells both in the presence and absence of the iron chelator BPS, indicating that regulation was at the level of transcription. This decrease in Fet3 expression was not observed in the *fep1∆* strain (Fig. 8c), suggesting that this process required Fep1-dependent repression. Iron-reductase activity (another target of Fep1) was also decreased in hypoxic GS115 cells but not in the *fep1∆* strain (Fig. 8d), strengthening the assumption that Fep1 activity can be modulated by oxygen.

As stated above, Fep1 WT would be able to switch between two conformations where the cluster is either bound by Cys102-Cys108-Cys117-Cys120 or by Cys65-Cys68. This hypothesis opens two possible scenarios: either the two clusters are assembled on the same dimer or each protein dimer can bind a cluster either at the central site or at the ZnF1 site. The Fe stoichiometry of Fep1 WT after aerobic reconstitution does not reach a value of 2 Fe/protein monomer, indicating that saturation of both sites on the same dimer is not achieved, at least in our conditions. However, the lineshape of the spectra of the aerobically reconstituted protein is consistent with an increase of both clusters, leaving both options open. A possible explanation for the existence of a mixture of two forms in aerobically purified (and reconstituted) Fep1 WT is that the two forms correspond to two local thermodynamic minima and that oxygen-induced conversion from the central Cys site to the ZnF1 site is kinetically slow *in vitro* unless a ‘catalyst’ is present. This suggests that *in vivo* the process could be assisted by other (as-yet unknown) factors, possibly proteins that interact with Fep1.

While this manuscript was in preparation, it was reported that *S. pombe* Fep1 coordinates iron via the ‘central’ cysteine residues directly and not as a Fe-S cluster^9^. This result is in contrast with our findings, and we have no straightforward explanation for this difference. One possibility is that a somewhat different mode of Fep1-dependent regulation is effective in the two yeast species, due to different iron requirements related to oxygen-dependent metabolic needs. *P. pastoris* is a Crabtree negative yeast, i.e. in normoxic conditions it exhibits a fully respiratory metabolism and this may lead to a higher demand for iron and explain the Fep1-dependent partial derepression of the iron regulon in normoxia compared to hypoxia.

## Conclusions

We present strong evidence by SAXS, optical and RR spectroscopy that Fep1, the iron-responsive transcriptional repressor from the methylotrophic yeast *P. pastoris*, is a dimer able to bind iron in the form of a [2Fe-2S] cluster. In the presence of oxygen Cys65-Cys68 of the first Zn finger can be recruited as alternative cluster ligands in Fep1 WT, suggesting that this transcriptional repressor might be both an iron and an oxygen sensor. At variance with the typical cluster conversion/degradation or redox modification employed by other iron or stress sensors, we propose that in the case of Fep1 oxygen can induce an alternative localization of the [2Fe-2S] cluster, causing a decreased affinity of this transcriptional repressor for DNA.

## Methods

### Yeast strains and media

*Pichia pastoris* strains GS115 (*his4*), JC304 (*ade1, his4*) and *fep1Δ* (*ade1, his4, fep1::ADE1*) were used. Yeast cells were grown at 30 °C in YPD or in MD minimal medium with the appropriate auxotrophic supplements. The bioavailable iron content of the medium was varied by addition of the membrane-impermeable iron chelator bathophenanthrolinedisulphonate (BPS) 80 μM, or FeCl_3_ 50–100 μM. Unless otherwise stated, exponentially growing cells (OD_600_ 1–2) were used for all experiments.

### Cloning of Fep1 by Inverse PCR

A 50-μg aliquot of GS115 genomic DNA, isolated by lysis with glass beads, was digested with either Sau3AI, TaqI or BsuRI for 10 min in a total volume of 200 μl. The digested DNA was extracted with an equal volume of phenol:chloroform:isoamyl alcohol (PCI 25:24:1), the aqueous phase was re-extracted with CI (24:1) and precipitated. The DNA was resuspended in 500 μl ligase buffer and self-ligated for 1 h at 16 °C with T4 ligase (Takara). The ligated DNA was extracted once with PCI followed by a CI extraction and precipitation. The circularised DNA was linearized by digestion with KpnI in a total volume of 50 μl at 37 °C overnight. The digested DNA was extracted with PCI, precipitated and resuspended in 50 μl of TE buffer. A 2-μl aliquot of the DNA sample was used in PCR reactions with primers Cys_for (gctcaggcttgcaaaggctgtccagc) and GATA6_rev (acacctaccgtcacctttacaagttcc). PCR conditions were as follows: initial denaturation at 95 °C for 5 min followed by 25 cycles at 94 °C for 30 s, 58 °C for 30 s and 72 °C for 2 min, followed by a final extension at 72 °C for 7 min. The PCR products obtained, about 1000 bp for Sau3AI, 450 bp for TaqI and 1100 bp for BsuRI were cloned in EcoRV-digested pBluescript II KS. Automated DNA sequencing was performed at Ylichron (ENEA, Italy).

### Expression of recombinant Fep1 in P. pastoris

Full-length Fep1 was cloned in the *P. pastoris* expression vectors pIB2 and pIB3^33^, under control of either the strong constitutive GAP promoter or of the low-level constitutive YPT1 promoter, respectively. N-terminal EGFP fusions and epitope-tagged Fep1 versions were produced by PCR. All Fep1 mutants were produced using the QuikChange II XL kit (Agilent) or by overlap-extension PCR and sequence-verified. Plasmids were linearized with SalI and used to transform electrocompetent *fep1∆* cells^3^. The presence of recombinant Fep1 was confirmed by PCR on genomic DNA from selected His^+^ colonies.

### Expression and purification of recombinant Fep1 in *E. coli*

Fep1 WT and cysteine mutants, either as the full-length protein or as the DNA-binding domain (amino acid residues 1–208) with a Flag tag at the N-terminus were cloned between NcoI and SalI in pET28a (Novagen) and expressed in *E. coli* BL21(DE3). Cells were induced with 0.1 mM IPTG for 4 h at 37 °C in medium supplemented with ZnSO_4_ 50 μM and Fe(NH_4_)SO_4_ 20 μM. Lysis of cells was carried out by sonication in 25 mM MOPS (pH 7.4) containing 120 mM NaCl, 1 mM EDTA, 0.5 mM DTT, 1 mM PMSF. Following centrifugation at 35000 rpm for 40 min, the supernatant was diluted 4-fold and applied to a CM-Sephadex C-50 column equilibrated in 25 mM MOPS (pH 7) containing 30 mM NaCl. After washing with 10 column volumes of equilibration buffer followed by 5 column volumes of MOPS buffer containing 150 mM NaCl, Fep1 was eluted with MOPS buffer containing 250 mM NaCl. All purification steps were carried out aerobically.

Anaerobic purification of Fep1 was carried out in a Belle Technology glove box (O_2_ < 4 ppm). The bacterial pellet was resuspended in lysis buffer, removed from the glove box for sonication (less than 5 min) and returned to the glove box. The lysate was diluted 1:1 with 25 mM MOPS buffer (pH 7), transferred to O-ring sealed centrifuge tubes and centrifuged outside of the glove box at 35000 rpm for 40 min. CM-Sephadex chromatography of the supernatant was performed anaerobically in the same conditions described above.

### Functional complementation assays

The activity of recombinant Fep1 was indirectly assayed through functional complementation on a *fep1∆* strain by measuring the iron-dependence of two systems known to be under control of Fep1, namely the ferroxidase Fet3 and the iron-reductase complex^3^. Fet3 oxidase activity was assayed by non-denaturing SDS-PAGE followed by equilibration of the gel in 10% glycerol/0.05% Triton X-100 for 30 min and then staining with 0.5 mg/ml *o*-dianisidine in 50 mM sodium acetate pH 5. Iron-reductase activity was assayed by placing a nitrocellulose filter directly onto colonies grown on MD + 200 μM FeCl_3_ plates and overlaying with a solution of 1 mM ferrozine, 1 mM FeCl_3_ in 50 mM sodium acetate, pH 5. Colonies with high ferrireductase activity turned purple almost immediately (within 1–2 min). For the liquid iron-reductase activity assay 100 μM FeCl_3_ and 1 mM ferrozine were added to cell extracts in 50 mM sodium acetate, pH 5 in the presence of 50 mM azide to inhibit Fet3. Absorbance of the Fe^2+^-ferrozine complex was measured at 562 nm.

### Electrophoretic mobility shift assay (EMSA)

To demonstrate specific DNA-binding activity for MBP-Fep1, EMSA binding reactions were carried out as previously described^3^. In brief, 1X binding buffer that contained 12.5 mM Hepes (pH 7.9), 75 mM NaCl, 4 mM MgCl_2_, 1 mM EDTA, 10% glycerol, 4 mM Tris-HCl (pH 7.9), 0.6 mM DTT, 1 μg of poly (dI-dC)_2_, 5 μM ZnSO_4_ and 5 μM FeCl_3_ unless otherwise stated, was used. Typically, 100–250 ng of affinity-purified MBP-Fep1 (41–192) WT or 4S was incubated for 20 min at 25 °C with 1 ng of α-^32^P-end-labeled double-stranded oligomers containing the GATAA site (fetgata2: ggcgattgaactgataattttactaaataagtggaa; fetgata1: ggattccacttatttagtaaaattatcagttcaat). The DNA-protein complex was separated from the free probe by electrophoresis onto a 6% native polyacrylamide gel in 0.25X TB (44.5 mM Tris, 44.5 mM borate) at 4 °C and 150 V for 2 h. The gel was then fixed, dried and the bands were detected by autoradiography.

### Biochemical methods

Yeast cell lysis with glass beads and membrane protein extraction were performed as described previously^3^. For Western blot analysis, the primary antibodies used were: monoclonal M2 and polyclonal anti-Flag (Sigma), polyclonal anti-GFP (Sigma) and polyclonal anti-MBP (Santa Cruz). After incubation with the appropriate HRP-conjugated secondary antibody, blots were developed with ECL Prime (GE Healthcare). Total protein content of membrane extracts and of purified protein samples was measured by the Bradford assay with bovine serum albumin as standard. Zinc and iron content of purified Fep1 was determined by ICP-AES; protein samples and standards were diluted in Chelex-treated 0.1 M HNO_3_ and analyzed on a Vista MXP Rad instrument (Varian). Iron content was also assayed spectrophotometrically with ferrozine. For the ferrozine assay, 500 μl of Chelex-treated 0.1 M HNO_3_ was added to 10-30 μl of sample (10-20 μM protein), incubated at room temperature overnight and pelleted for 5 min at 15000g. To the supernatant were then added 20 μl of 10 mM ferrozine, 20 μl of 75 mM ascorbic acid and 170 μl of an ammonium acetate saturated solution, incubated for 20 min at room temperature. Iron content was then determined at 562 nm (ε_562_ 27900 M^−1^ cm^−1^). Acid-labile sulfur was measured according to published methods^34^,^35^. Glutathione content was assessed by the DTNB-GSSG reductase recycling assay^11^.

Reconstitution of Fep1 (1–208) was performed by addition of FeCl_3_ and Na_2_S in the presence of 0.5–1 mM TCEP in aerobic and anaerobic conditions; after 10 min incubation no further color changes were noted, samples were centrifuged and spectra were recorded. For metal content analysis unbound iron and sulfide were removed by gel-filtration on a PD-10 column (GE Healthcare) equilibrated in 25 mM MOPS (pH 7) containing 250 mM NaCl.

Analytical gel-filtration was performed on a Superdex-75 (10/300) column (GE Healthcare) equilibrated in 25 mM MOPS (pH 7) containing 150 mM NaCl. The Superdex-75 column was calibrated with conalbumin (M_r_ 75 kDa), ovalbumin (M_r_ 43 kDa), carbonic anhydrase (M_r_ 29 kDa) and RNase A (M_r_ 13.7 kDa).

### Spectroscopic methods

Optical spectra were recorded at 25 °C with a Perkin Elmer Lambda 20 spectrophotometer using a 1-cm cuvette and a Cary 60 (Agilent Technologies) using a 5-mm NMR tube at a scan rate of 300 nm/min.

Resonance Raman (RR) spectra were obtained at room temperature using a 5-mm NMR tube by excitation with the 413.1 nm line of a Kr^+^ laser (Coherent Innova 300 C, Santa Clara, CA). Backscattered light from a slowly rotating NMR tube was collected and focused into a triple spectrometer (consisting of two Acton Research SpectraPro 2300i and a SpectraPro 2500i in the final stage with a 3600 grooves/mm grating) working in the subtractive mode, equipped with a liquid nitrogen-cooled CCD detector. A spectral resolution of 1 cm^−1^ was calculated theoretically on the basis of the optical properties of the spectrometer for a 3600 grating. The RR spectra were calibrated with CCl_4_ and *n*-pentane as standards to an accuracy of +/− 1 cm^−1^ for intense isolated bands. Absorption spectra were measured both prior to and after RR measurements to ensure that no degradation had taken place under the experimental conditions used. All spectra were baseline corrected. Protein concentrations were 0.3–0.4 mM Fe.

Solution small-angle X-ray scattering (SAXS) measurements were performed at the beamline BM29 of the European Synchrotron Radiation Facility (ESRF). The synchrotron was operating in 16-bunch mode giving a beam intensity of between 85 and 90 mA. Data collection was performed at 12 keV with BsxCuBE, exposing each frame for 2 sec, with continuous flow in the capillary; three dilutions of each sample were measured at 293 K. The analyzed spectra were the result of 5–10 averaged single curves (collected taking care to eliminate curves with radiation damage). Data analysis was performed with the ATSAS suite^25^.

## Additional Information

**How to cite this article**: Cutone, A. *et al.* *Pichia pastoris* Fep1 is a [2Fe-2S] protein with a Zn finger that displays an unusual oxygen-dependent role in cluster binding. *Sci. Rep.* **6**, 31872; doi: 10.1038/srep31872 (2016).

## Supplementary Material

Supplementary Information
