# Supplementary material for: Pichia pastoris Fep1 is a [2Fe-2S] protein with a Zn finger that displays an unusual oxygen-dependent role in cluster binding
Source: Sci Rep. 2016 Aug 22;6:31872. doi: 10.1038/srep31872 (PMC4992955; doi:10.1038/srep31872)
Supplement: Supplementary Information [file srep31872-s1.pdf]

*Pichia pastoris* Fep1 is a [2Fe-2S] protein with a Zn finger that displays an unusual oxygen-dependent role in cluster binding.

Antimo Cutone[1] Barry D. Howes[2], Adriana E. Miele[1], Rossella Miele[1], Alessandra Giorgi[1], Andrea Battistoni[3], Giulietta Smulevich[2], Giovanni Musci[4] and Maria Carmela Bonaccorsi di Patti[1*]

[1]Dip. Scienze Biochimiche 'A. Rossi Fanelli', Sapienza Università di Roma, Roma, Italy
[2]Dip. Chimica 'Ugo Schiff', Università di Firenze, Sesto Fiorentino (FI), Italy
[3]Dip. Biologia, Università di Roma Tor Vergata, Roma, Italy
[4]Dip. Bioscienze e Territorio, Università del Molise, Pesche, Italy

*To whom correspondence should be addressed: M. Carmela Bonaccorsi di Patti, Dip. Scienze Biochimiche 'A. Rossi Fanelli', Sapienza Università di Roma, P. le Aldo Moro 5, 00185 Roma, Italy. Tel.: +390649917573; Fax: +390649917566; E-mail: mariacarmela.bonaccorsi@uniroma1.it

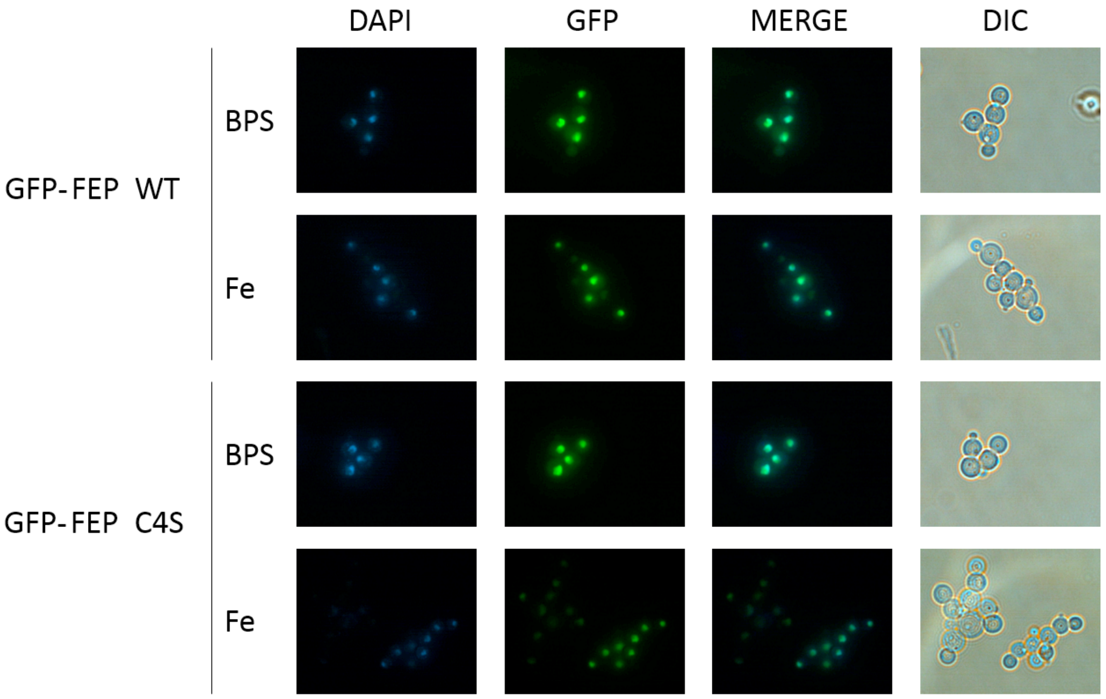


**Fig S1**. **Fluorescence microscopy analysis of the subcellular localization of GFP-Fep1 WT and 4S.** *P. pastoris fep1*Δ cells expressing GFP-Fep1 WT and 4S were grown in YPD + BPS 80 µM or YPD + Fe 100 µM. The cells were fixed and nuclei were stained with Hoechst 33342 (5 µg/ml). Cells were visualized using a Leica DM500B microscope equipped with a 100x oil immersion objective and a DFC340fx camera. Images were processed with ImageJ.

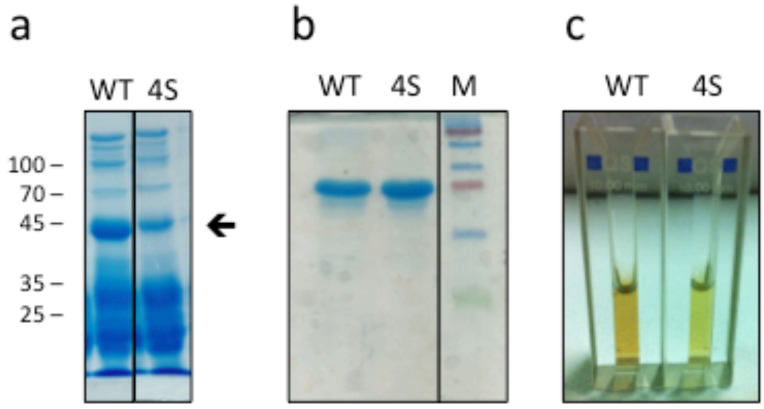


**Fig S2. SDS-PAGE analysis of purified Fep1**. **a**) Full-length Fep1. Lane 1: WT, lane 2: 4S, the arrow indicates the position of the full-length protein. **b**) Fep1 (1-208). Lane 1: WT, lane 2: 4S, lane 3: $M_r$ marker (the lower red band corresponds to $M_r$ 25 kDa). **c**) Fep1 (1-208) WT and 4S.

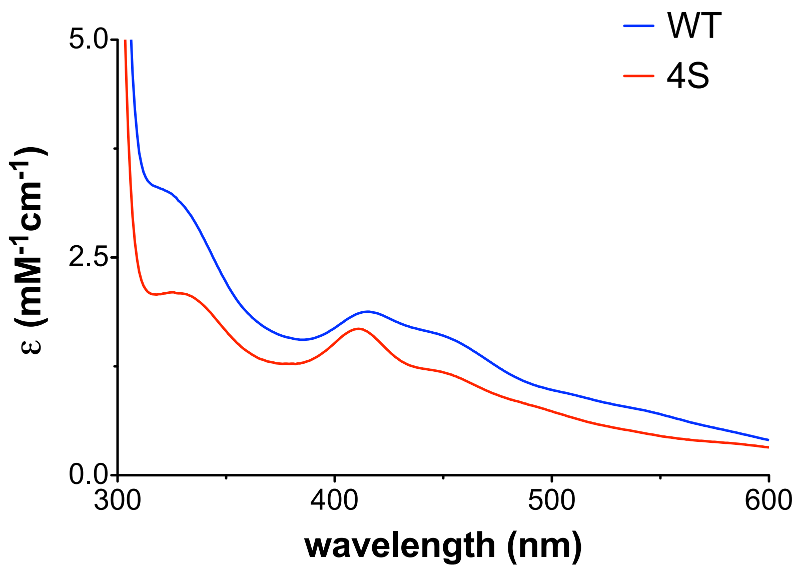


**Fig S3. Absorption spectra of full-length Fep1 purified aerobically**. Spectrum of WT is blue and 4S is red. Molar extinction coefficients (ε) are based on the concentration of iron. The spectra are identical to those of the corresponding DNA-binding domains (1-208) shown in Fig. 3a.

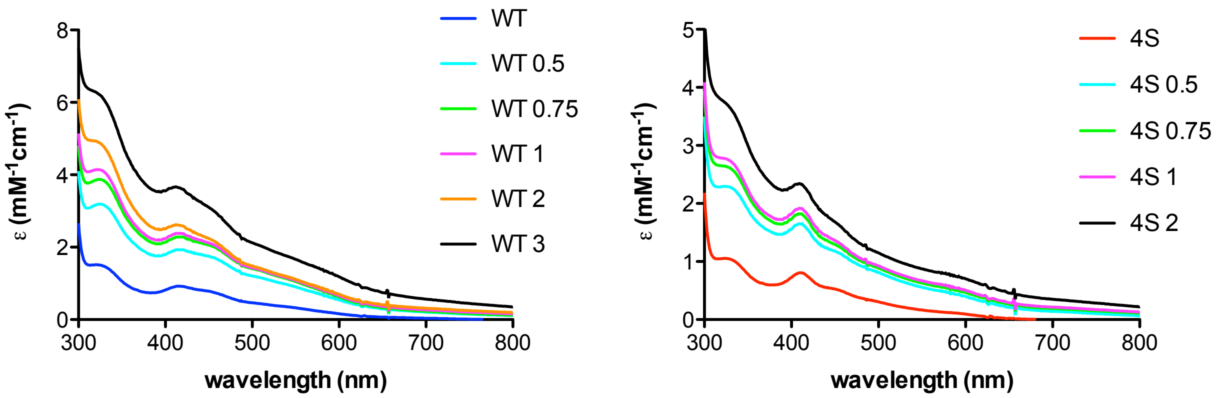


**Fig S4**. **Reconstitution of Fep1**. Absorption spectra of Fep1 (1-208) WT (left panel) and 4S (right panel) before and after aerobic reconstitution with the indicated excess of $FeCl_3$ and $Na_2S$ in the presence of TCEP 0.5 mM. Molar extinction coefficients (ε) are expressed per Fep1 monomer.

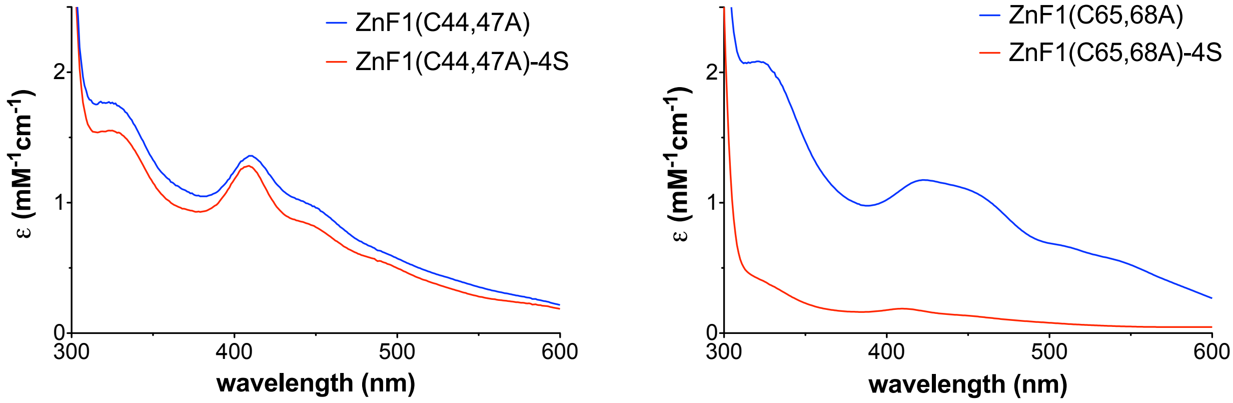


**Fig S5**. **Absorption spectra of Fep1 (1-208) Zn finger 1 mutants purified aerobically**. Left panel: ZnF1(C44,47A) lacking Cys44 and Cys47; right panel: ZnF1(C65,68A) lacking Cys65 and Cys68. Mutants in a WT background are shown as blue lines, those in a 4S background are shown as red lines. Molar extinction coefficients (ε) are expressed per Fep1 monomer.

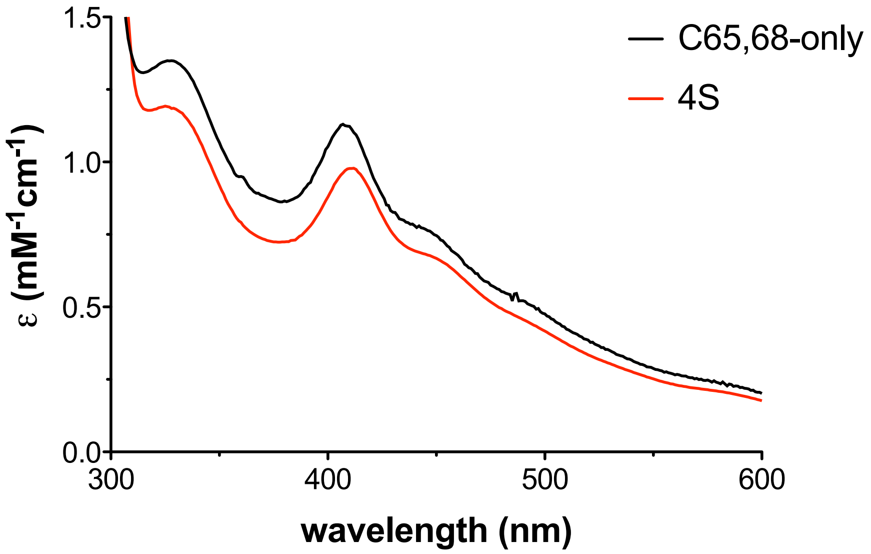


**Fig S6**. **Absorption spectra of Fep1 (1-208) cysteine mutants purified aerobically**. C65,68-only (black), for comparison a 4S spectrum (red) is shown. Molar extinction coefficients (ε) are expressed per Fep1 monomer.

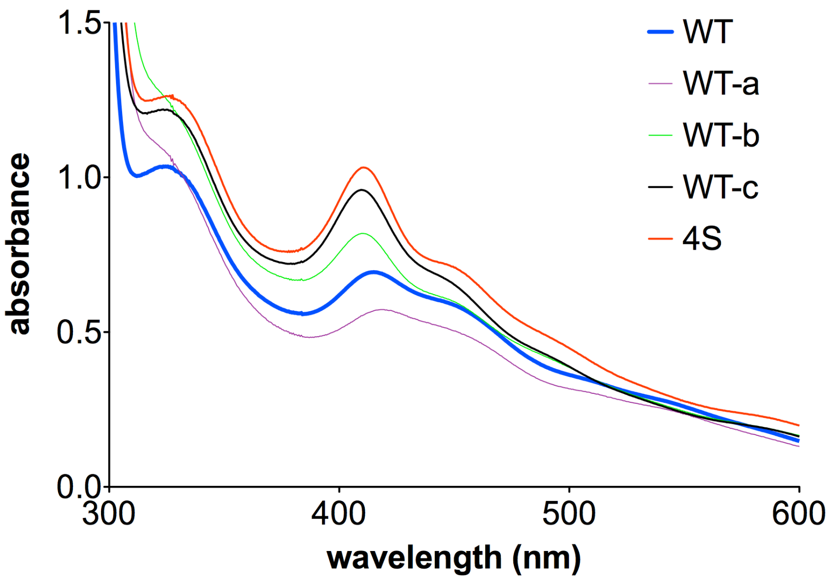


**Fig S7**. **Absorption spectra of different samples of Fep1 (1-208) WT obtained aerobically and of a representative sample of 4S (red)**. The thick blue line shows a typical WT sample, the black spectrum (WT-c) is of a sample of WT with 4S features (see text for details). The spectra are arbitrarily scaled to show the lineshape differences in the 400-450 nm region.

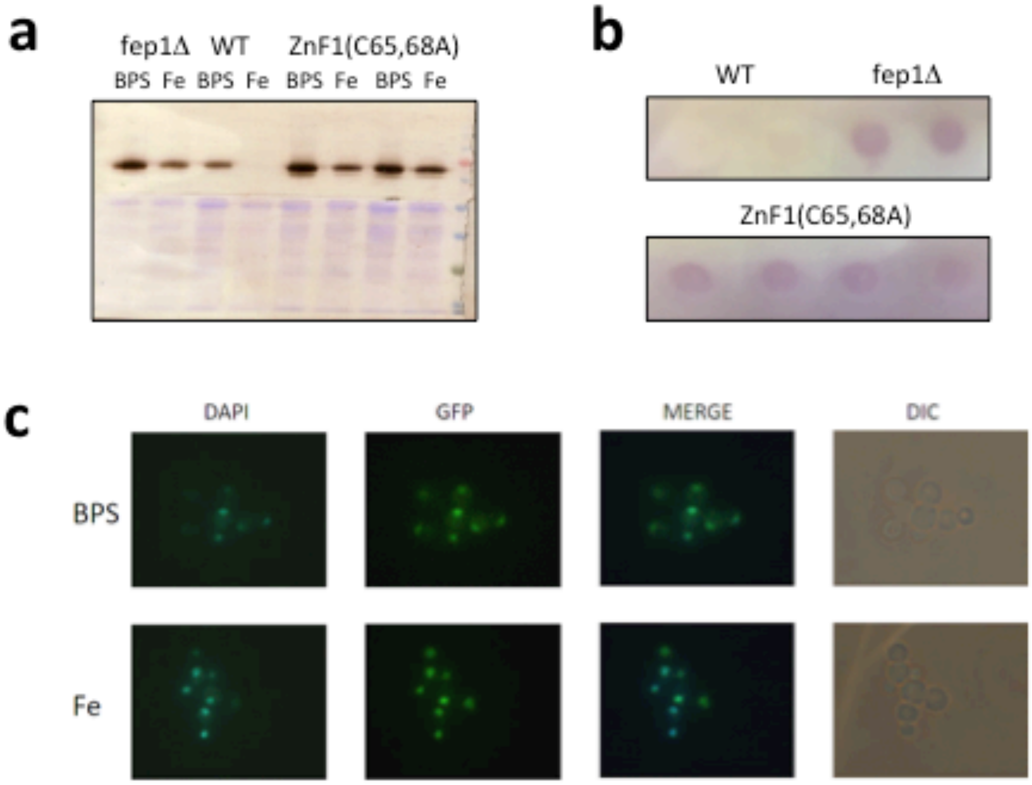


**Fig S8. Fep1 ZnF1(C65,68A) does not complement *P. pastoris* strain *fep1Δ*. a**) Non-denaturing SDS-PAGE analysis of Fet3 oxidase activity of *fep1Δ*, Fep1 WT, Fep1 ZnF1(C65,68A) grown in YPD + BPS 80 µM or YPD + $FeCl_3$ 100 µM. The upper part of the gel is stained with *o*-dianisidine to show Fet3 oxidase activity; the lower part of the gel is stained with Coomassie Blue to show total protein content of samples loaded on the gel. **b**) *In vivo* iron-reductase assay of *fep1Δ*, Fep1 WT, Fep1 ZnF1(C65,68A). Iron-reductase activity was assayed by placing a nitrocellulose filter directly onto colonies grown on MD + $FeCl_3$ 200 µM plates and overlaying with a solution of 1 mM ferrozine, 1 mM $FeCl_3$ in 50 mM sodium acetate, pH 5. **c**) Fluorescence microscopy analysis of the subcellular localization of GFP-Fep1 ZnF1(C65,68A) grown in YPD + BPS 80 µM or YPD + Fe 100 µM. The cells were fixed and nuclei were stained with Hoechst 33342 (5 µg/ml). Cells were visualized using a Leica DM500B microscope equipped with a 100x oil immersion objective and a DFC340fx camera. Images were processed with ImageJ.

**Table S1**. Resonance Raman Fe-S stretching frequencies for [2Fe-2S] clusters

| Mode $D_{2h}$[a] | This work[g] | | | | Ado[b] | PuFd[b] | CpFd[c] | PpFd[c] | **AvIscU[d]** | **Av NifU-1[e]** |
|---|---|---|---|---|---|---|---|---|---|---|
| | WT/4S[h] | Z1*[h] | WT/Z1*[i] | 4S[i] | | | | | | |
| $B_{2u}^{b}$ | | | 415vw | | 421 | 426 | 404 | 426 | **425** | |
| $A_{g}^{b}$ | 393s | 391s | 391s | 394vs | 393 | 395 | 387 | 400 | **406** | **401** |
| $B_{3u}^{b}$ | | | | | 349 | 367 | 366 | 350 | **367** | **364** |
| $B_{1u}^{t}$, $B_{2g}^{t}$ [f] | 345[f]m | 345[f]s | 347[f]s | 347[f]w | 341 | 357 | 353 | 344 | | |
| $A_{g}^{t}$ | 325s | 327s | 328s | 325vs | 329 | 339 | 335 | 338 | **353** | **349** |
| $B_{1g}^{b}$ | 320s | 320s | 320s | 320s | 317 | 329 | 313 | 320 | **328** | **320** |
| $B_{3u}^{t}$ | 284w | 284w | 284w | 284w | 291 | 282 | 290 | 291 | **296** | **294** |

[a]Symmetry labels under $D_{2h}$ symmetry for a $2Fe2S^{b}4S^{t}$ unit; t and b refer to terminal and bridging S atoms, respectively. [b]Assignments from [1] for adrenodoxin and *P. umbilicalis* ferrodoxin. [c]Assignments from [2] for *C. pasteurianum* and *P. putida* ferrodoxins. [d]Assignments from [3] for *A. vinelandii* IscU. [e]Assignments from [4] for *A. vinelandii* NifU-1. [f]These modes are only enhanced for excitation at 406/413 nm [2]. Due to the very strong fluorescence observed for the Fep1 proteins at visible wavelength excitation (where these modes should be absent), the assignment of this band to a $B_{3u}^{b}$ mode cannot be completely excluded. See text. [g]vs, very strong; s, strong; m, moderate; w, weak; vw, very weak. [h]Aerobically prepared samples. [i]Anaerobically prepared samples. The two extreme right columns (bold) correspond to examples of [2Fe-2S] clusters with incomplete cysteinyl coordination, as demonstrated by the expected upshift of the $A_{g}^{t}$ and $B_{3u}^{t}$ Fe-S modes (shown in red) compared to those of [2Fe-2S] clusters with complete cysteinyl coordination (all other columns). *Abbreviation: Z1, ZnF1(C65,68A).

**Table S2**: Iron, acid-labile sulfur and zinc content of Fep1 (1-208) WT and 4S [a]

| **Fep1 (1-208)** | **Fe/protein** | **S/protein** | **Zn/protein** |
|---|---|---|---|
| WT[b] | 0.20 (0.01) | 0.30 (0.05) | 1.40 (0.13) |
| WT after reconstitution | 1.19 (0.05) | 1.02 (0.14) | 0.95 (0.07) aerobic<br>1.33 (0.14) anaerobic |
| 4S | 0.18 (0.01) | 0.28 (0.02) | 1.46 (0.11) |
| 4S after reconstitution | 1.01 (0.08) | 0.66 (0.10) | 0.95 (0.10) |

[a] The results are the mean (±SE) of 3-8 independent experiments.
[b] Metal content (Fe/S/Zn) of anaerobically purified WT was similar to that of the aerobically purified protein.

**Table S3**. Iron and acid-labile sulfur content of Fep1 (1-208) cysteine mutants purified aerobically[a]

| **Fep1 (1-208)** | **Fe/protein** | **S/protein** |
|---|---|---|
| noZnF1 | 0.19 | 0.51 |
| noZnF1-4S | 0.02 | 0.09 |
| noZnF2 | 0.24 | 0.35 |
| noZnF2-4S | 0.26 | 0.32 |
| noZnFs | 0.36 | 0.53 |
| noZnFs-4S | n. d. | 0.03 |
| ZnF1(C44,47A) | 0.17 | 0.08 |
| ZnF1(C44,47A)-4S | 0.17 | 0.22 |
| ZnF1(C65,68A) | 0.22 | 0.12 |
| ZnF1(C65,68A)-4S | 0.03 | 0.17 |
| C65,68-only | 0.14 | 0.27 |

[a] Representative results for single protein samples are reported. Variation in iron and acid-labile sulfur content was about 10% among different samples of the same mutant. n. d.: not detected.

## References


1. Han, S., Czernuszewicz, R.S., Kimura, T., Adams M.W.W. & Spiro, T.G. $Fe_2S_2$ protein resonance Raman-spectra revisited; Structural variations among adrenodoxin, ferredoxin, and red paramagnetic protein. *J Am Chem Soc* **111**, 3505–3511 (1989).
2. Fu, W., Drozdzewski, P.M., Davies, M.D., Sligar, S.G. & Johnson, M.K. Resonance Raman and magnetic circular dichroism studies of reduced [2Fe-2S] proteins. *J Biol Chem* **267**, 15502–15510 (1992).
3. Agar, J.N. *et al.* IscU as a scaffold for iron-sulfur cluster biosynthesis: sequential assembly of [2Fe-2S] and [4Fe-4S] clusters in IscU. *Biochemistry* **39**, 7856–7862 (2000).
4. Yuvaniyama, P., Agar, J.N., Cash, V.L., Johnson, M.K. & Dean, D.R. NifS-directed assembly of a transient [2Fe–2S] cluster within the NifU protein. *Proc Natl Acad Sci USA* **97**, 599–604 (2000).
